# Adolescent physical activity profiles as determinants of emerging adults’ physical activity

**DOI:** 10.1186/s12966-025-01732-9

**Published:** 2025-03-25

**Authors:** Mathieu Bélanger, Marie-Andrée Giroux, Pierre Philippe Wilson Registe, François Gallant, Salma Jemaa, Pierre Faivre, Daniel Saucier, Saïd Mekari

**Affiliations:** 1 Faculty of Medicine and Health Sciences, Université de Sherbrooke, Sherbrooke, Québec Canada; 2 IMPACTS Laboratory, Centre de Formation Médicale du Nouveau-Brunswick, Moncton, New Brunswick, Canada; 3 Vitalité Health Network, Moncton, New Brunswick, Canada; 4 Dalhousie University, Halifax, NS Canada

**Keywords:** Context of physical activity, Longitudinal study, Trajectories, Life course, Tracking

## Abstract

**Background:**

Although adolescent physical activity (PA) tracks into adulthood, it is unclear if the context of PA practiced during adolescence predicts adult PA. We previously identified five adolescent PA profiles and aimed to assess associations between these profiles and levels of PA in emerging adulthood.

**Methods:**

Using data from the first 8 years of the MATCH study, when participants were 11 to 18 years, we identified five adolescent PA profiles: “non-participants” (9% of the sample), “dropouts” (30%), “active in unorganized PA” (19%), “active in organized PA” (27%), and “active through a variety of PA” (15%). The same participants reported their PA level (IPAQ) 2.5, 3.5, 4.5, and 5.5 years later when they were emerging adults (20, 21, 22, and 23 years). The adolescent PA profiles were included in a mixed-distribution two parts model as predictors of i) the probability of reporting any PA during emerging adulthood, and ii) the PA level of emerging adults reporting PA.

**Results:**

Being categorized as “non-participant” or “dropout” during adolescence was associated with similar likelihoods of reporting PA and PA level during emerging adulthood. In contrast with “non-participants”, those in the “active-unorganized” (OR, 95% CI: 2.8, 2.1–3.8), “active-organized” (2.4, 1.7–3.2), and “active-variety” (3.7, 3.3–5.1) were considerably more likely to report any PA during emerging adulthood. Among emerging adults who reported some PA, those with an adolescent profile of “active-unorganized”, “active-organized” and “active-variety” had higher PA levels than “non-participants” (all *p* < 0.01).

**Conclusion:**

Profiles of PA participation during adolescence represent strong predictors of PA in emerging adulthood. Promoting participation in various types of PA during adolescence is key to preventing low PA among emerging adults.

**Supplementary Information:**

The online version contains supplementary material available at 10.1186/s12966-025-01732-9.

## Background

Physical activity levels decline significantly during adolescence and often remain low into adulthood [1]. It is therefore in emerging adulthood, when people are in their 20 s, when physical activity typically reaches its lowest levels [2]. A potentially important determinant of physical activity participation of emerging adults includes early experiences with specific types of physical activities [3, 4]. Studies suggest there is variation across physical activity types regarding the likelihood that they are sustained over time. For example, studies that tracked physical activity throughout adolescence identified that activities that are unorganized and that can be practiced individually are more likely to be maintained over time than organized and group-based activities, respectively [5, 6]. Moreover, one group identified that whereas overall physical activity level during adolescence does not predict emerging adults’ physical activity, engagement in unorganized physical activity during adolescence, either through leisure or transportation activities, was positively associated with emerging adults’ physical activity levels [7]. Similarly, another study reported that it was the number of years of participation in sports and running during adolescence that better predicted higher levels of emerging adult physical activity, while adolescents’ participation in fitness and dance activities was not a determinant of future physical activity level [4]. Further, although they did not compare future physical activity levels across types of activities practiced in adolescence, Kjonniksen, Anderssen and Wold [8] documented that the number of years of participation in organized sports during adolescence can also be a positive determinant of emerging adults overall physical activity level. Therefore, it is unclear which type or context of exposure to physical activity during adolescence is the most likely to lead to higher physical activity levels in emerging adulthood.

Correspondingly, recent studies suggested that the description of adolescent physical activity behavior should go beyond a simple representation of their level of physical activity, but instead should account for multiple characteristics of the behavior, such as involvement in organized and unorganized physical activity as well as the extent to which one takes part in different activities [9]. However, most previous studies which characterized distinct longitudinal physical activity or sport participation profiles throughout adolescence did so using only one dimension of physical activity [10–12]. This restricted view of physical activity precludes our understanding of the evolution of the behavior during adolescence and limits our knowledge of how distinct adolescent physical activity profiles influence adult physical activity. Nevertheless, a recent study by Gallant et al. identified naturally occurring longitudinal profiles of adolescent participation in physical activity that account for physical activity level, engagement in organized sports and participation in unorganized physical activity [13]. Specifically, these authors applied multi-trajectory latent class analyses to 24 cycles of data cumulated over 8 years to find that adolescents generally fall into one of five general profiles of sport and physical activity participation, which are: 1) non-participants (those who consistently display low or no participation in sports and physical activity), 2) drop-outs (those who enter adolescence as somewhat active, but drop out of sports and physical activity rapidly), 3) active through organized physical activity (those who remain active throughout adolescence due to their engagement in organized physical activity only), 4) active through unorganized physical activity (those who remain active throughout adolescence exclusively through unorganized activities), and 5) highly active (those who report participation in a variety of organized and unorganized sports and physical activity throughout adolescence). Knowing how these various profiles relate to future physical activity levels would help identify physical activity behaviors to promote during adolescence.

One hypothesis put forward by various models of sport and physical activity participation development is that exposure to a larger variety of types of physical activity during the development years helps develop the foundational skills and aptitudes required to sustain long-term physical activity participation [14]. Indeed, physical literacy, which denotes the ability to move with competence and confidence in a wide variety of physically challenging situations [15], is often regarded as the “cornerstone” for an active lifestyle [16]. As such, it is possible that belonging to a physical activity profile that incorporates a variety of organized and unorganized physical activities during adolescence better prepares adolescents to maintain physical activity when they become emerging adults. Therefore, it can be hypothesized that people who were the most physically active during adolescence, particularly those who took part in a greater variety of activities, would also be among the most active in early adulthood. The goal of this study was to estimate the association between adolescent physical activity profiles and emerging adulthood physical activity levels.

## Methods

We used data from the Monitoring Activities of Teenagers to Comprehend their Habits (MATCH) study, an ongoing longitudinal study of 929 youth from New Brunswick, Canada [17]. Briefly, participants were recruited from 17 schools selected purposively to represent students of different socioeconomic status, from French and English backgrounds, and those living in rural and urban environments. Participants reported their free-time physical activity every four months during the school years (from the ages of 10–11 to 17–18), for a total of 24 data collection cycles. They took part in a 25th cycle 2–3 years after graduation from high school, followed by three additional cycles at one-year intervals (cycles 26, 27, and 28). These four cycles took place between December and May of 2020–21, 2021–22, 2022–23, 2023–24, when participants were 20, 21, 22, and 23 years on average. Data collection took place through online questionnaires using the LimeSurvey platform. Participants could answer in English or French and were able to review their answers before submitting them. Data were stored on secured institutional servers. The MATCH study received Ethics Approval from the Comité d’Éthique de la Recherche du Centre Hospitalier de l’Université de Sherbrooke. Participants provided consent in completing the questionnaire and received a monetary compensation after completing the questionnaires at cycles 25–28.

### Study Variables

#### Adolescent moderate-to-vigorous physical activity (MVPA)

At cycles 1–24, participants were provided with the following definition of MVPA “*Physical activity is any activity that increases your heart rate and makes you get out of breath some of the time. Physical activity can be done in sports, playing with friends, or walking to school. Some examples of physical activity are running, brisk walking, rollerblading, biking, dancing, skateboarding, swimming, soccer, basketball, hockey, and skiing.*” Participants were then asked to report the number of days they engaged in MVPA for at least 60 min in [1] a typical week, and [2] in the past week. Response options ranged from 0 to 7 (days) and the two items were averaged to create an overall MVPA score [18]. Using this approach in previous MATCH analyses yielded estimates similar to those obtained through objective measures among a representative sample of same-aged Canadian youth [19]. This measure has acceptable test–retest reliability (ICC = 0.77) and is associated with accelerometer-measured MVPA (*r* = 0.40) [18].

#### Adolescent organized and unorganized physical activity

At each data collection cycle, participants reported their involvement in 36 different physical activities in the past 4 months using a checklist. For each physical activity, participants reported the frequency of participation (*i.e., never, once per month, 2–3 times per month, once per week, 2–3 times per week, 4–5 times per week, almost every day)* and with whom they most often took part in that activity (*i.e. alone, with friends, with parents/siblings, or with an organized group or team)*. Using a previously validated methodology for the current study [20], each activity was classified as organized or unorganized depending on with whom participants reported most often practicing the activity. While seven activities were always classified as unorganized (*i.e., home exercises, trampoline, games (chase, tag, hide and seek, skipping rope, weight training, and indoor and outdoor chores)*, the remaining activities were classified as organized if participants reported involvement with ‘an organized group or team’. Otherwise, the activities were classified as unorganized. The number of organized and unorganized physical activities reported were computed for every participant at every survey cycle.

#### Adolescent physical activity profiles

We previously identified adolescent physical activity profiles using cycles 1–24 measures of MVPA, number of organized physical activities and number of unorganized physical activities described above [13]. Briefly, these profiles were identified through group-based multi-trajectory models which identifies latent classes through an application of finite mixture modeling [21]. Specifically, the profiles represent groupings of participants who displayed similar trends in their levels of engagement in MVPA, number of organized physical activities and number of unorganized physical activities throughout adolescence. Per current recommendations [21, 22], the multi-trajectory model selection of longitudinal physical activity participation patterns was informed by the Bayesian Information Criterion (BIC), mismatch (i.e., the difference between the model estimated class proportions and class membership proportions), average posterior probability (i.e., probability that participants are assigned to the most likely class based on their developmental trajectory), and relative entropy (a standardized measure for classification uncertainty accounting for the entire sample by group posterior probabilities). We tested models that included from two to six distinct profiles of participants. Only participants with at least three cycles of data during adolescence were included as a minimum of three time points are required to be able to document a trajectory with a quadratic function [21]. In the end, the 893 participants with at least three cycles of data were classified in one of five adolescent physical activity participation profile. Based on the average trajectories of participation in MVPA, organized, and unorganized physical activities of participants in each profile, we labeled these profiles as nonparticipants (*n* = 76), dropouts (*n* = 265), active in unorganized physical activity (*n* = 172), active in organized physical activity (*n* = 243) and active in a variety of physical activity (*n* = 137). As suggested by the names attributed to the profiles, the non-participants presented consistently low levels of participation in any type of physical activity throughout adolescence; the drop-outs displayed some involvement in physical activity in early adolescence, but then dropped out of all physical activities rapidly; the active through organized physical activity reported consistently high levels of MVPA throughout adolescence since they maintained involvement in organized physical activity, but not in unorganized physical activity; the active through unorganized physical activity also remained active throughout adolescence, but this was entirely attributable to the maintenance of involvement in unorganized physical activity as they consistently reported very little participation in organized physical activity; and the highly active were characterized by engagement in a high level of MVPA as well as in both organized and unorganized physical activity throughout adolescence.

#### Adult physical activity

In cycle 25, 26, 27, and 28, participants self-reported their weekly moderate-to-vigorous physical activity (MVPA) level using the International Physical Activity Questionnaire – Short Form (IPAQ-SF) [23]. The IPAQ-SF estimates time spent in walking, moderate-, and vigorous-intensity physical activity in the past 7 days. For each intensity level, participants report the typical number of days per week and usual time spent doing activities on those days. IPAQ-SF data were processed using the standardized protocol available online (https://sites.google.com/view/ipaq/score). Total physical activity level was estimated by multiplying time spent at each activity intensity by the number of days it was reported [23]. Each activity intensity was weighted by its respective energy expenditures expressed in metabolic equivalent of task (MET)-minutes per week, where one MET is equivalent to oxygen consumption while sitting quietly at rest (3.5 ml/kg/min)[23].

### Covariates

We built a directed acyclic graph (DAG) to identify variables to account for in the analyses [24]. The starting point of this DAG were all manuscripts included in literature reviews by Oja et al. 2015 [25] and Batista et al. 2019 [26]. All adjustment variables accounted for in these manuscripts of a longitudinal association between previous physical activity levels and adult physical activity were included in our DAG. This DAG allowed to determine that the minimal adjustment set for estimating the total effect of adolescent physical activity participation profile on adult physical activity level includes gender, ethnic background, socioeconomic status during adolescence, rurality, familial history of physical activity, and disability (Appendix 1). We omitted disability from the analyses since no participant reported it in an open field section included in all survey cycles which invited participants to report “circumstances or conditions that prevented them from taking part in physical activity”. We also did not have information on participants’ familial history of physical activity so could not include a variable to represent this concept. Data for all other potential confounders were included. Specifically, participants reported their gender at survey cycles 25–28 as: man, woman, trans man, trans woman, non-binary, or not listed. For ethnic background, participants were asked to select all cultural/ethnic backgrounds that applied to them from a list that included: White (British, French, Italian, Portuguese, Ukrainian, Russian, Israeli), Aboriginal (First Nations, Inuit, Métis, non-status Indian), Black, Chinese, Japanese, South Asian (East Indian, Pakistani, Bangladeshi, Sri Lankan), Southeast Asian (Cambodian, Indonesian, Laotian, Vietnamese, Malaysian), West Asian (Afghan, Iranian), Latin American, Central American, South American (Mexican, Brazilian, Chilean, Guatemalan, Venezuelan, Colombian, Argentinian, Salvadorian, Costa Rican), Arabic or Other. For the socioeconomic status of participants, we matched their postal codes to the 2011 mean income of individuals (> 15 years) in their neighborhood, as per the National Household Survey census data. For rurality, we followed recommendations by Statistics Canada such that participants were classified in the rural category if the postal code they reported during adolescence is in a census subdivision or municipality where there are < 10,000 inhabitants, whereas postal codes associated to at least 10,000 inhabitants were considered urban [27].

### Statistical analyses

Data are described using frequencies, proportions, means and standard deviations. As noted in other studies using the IPAQ-SF, a substantive proportion of participants presented zero values on our measure of MVPA at cycle 25, 26, 27, and 28 (when participants were 20, 21, 22 and 23 years old) [28–30]. The distribution of MVPA values for other participants followed a log-normal distribution. Therefore, we developed a mixed-distribution model made up of two parts using the approach suggested by Tooze et al. (2002) for analyzing repeated measures data that are semi-continuous and which present considerable clumping at zero [31]. The first part was a logistic model estimating differences in the probability of reporting some MVPA (versus no MVPA) during emerging adulthood across participants with different adolescent physical activity profiles. The second part used a log–normal function to model the level of MVPA among emerging adult participants reporting some MVPA as a function of their adolescent physical activity profiles. This mixed-effects mixed-distribution model was developed with the SAS-based (version 9.4) MIXCORR macro. Final model selection (uncorrelated or correlated model) was determined by considering if the covariance was significant, by comparing AIC values and by computing a likelihood ratio test. Both parts of the model included adjustments for time (survey cycle 25, 26, 27, or 28), gender (man, woman, or other), ethnic background (white or other), mean income of individuals in participants’ neighborhood during adolescence, and rurality during adolescence (rural or urban). Computing this model with 500 participants would provide 80% power to detect a small effect size and 92% power to detect a medium effect size (Cohen’s f^2^ of 0.02 and 0.15, respectively) at alpha = 0.05. Prior to these analyses, we used a Cochran-Armitage test to assess if there was a trend in the proportion of participants reporting no physical activity over the years. To assess if there was a trend in the level of physical activity among participants who reported participation in some physical activity over the years, we ran a simple linear regression with the log of IPAQ as the outcome and survey cycle as the exposure. Finally, we also ran Tukey’s multiple comparisons to identify pairs of adolescent physical activity participation profiles that presented statistically different levels of MVPA in emerging adulthood.

## Results

Of the 929 participants who took part in the MATCH study during adolescence, 530 were retained for the current analysis as they provided the data required in at least one of the four survey cycles occurring when they were 20, 21, 22 or 23 years old, respectively. Of these, 62 (12%) participated in one survey, 64 (12%) in two surveys, 106 (20%) responded to three surveys, and 298 (56%) took part in all four surveys that took place in emerging adulthood. These participants had also taken part in a median of 17 (quartile 1 = 11, quartile 3 = 20) of the 24 adolescent survey cycles. Approximately two thirds of these participants were women, half grew up in a rural region and 95% were white (Table 1). When they were adolescents, 7% of participants included in the analysis were described as non-participants in physical activity, 30% were characterized by presenting a dropout in their physical activity, 17% were physically active because of their involvement in unorganized activities, 30% were active through organized physical activities, and 15% were active through engagement in a variety of physical activities. There was no relationship between the adolescent physical activity profile and the number of survey cycles participated in during emerging adulthood as 74% to 77% of participants in each group responded to at least 3 of the 4 adult surveys. The proportion of study participants reporting taking part in no physical activity more than doubled throughout the four years of emerging adulthood (*p* for trend < 0.001), but the physical activity level of those who reported taking part in some physical activity increased by over 500 MET-minutes per week over the same period (*p* for trend = 0.003).

**Table 1:** Characteristics of study participants retained for the analyses (*n *= 530)

	**Cycle 25**	**Cycle 26**	**Cycle 27**	**Cycle 28**
	*n* = 467	*n* = 407	*n* = 405	*n* = 418
**Gender, n (%)**				
Woman	287 (61.5)	261 (64.1)	254 (62.7)	265 (63.4)
Man	173 (37.0)	134 (32.9)	140 (34.6)	142 (34.0)
Other	7 (1.5)	12 (3.0)	11 (2.7)	11 (2.6)
Mean years (SD)	20.0 (0.7)	21.0 (0.6)	21.9 (0.6)	23.0 (0.6)
**Income during adolescence**				
Mean Canadian dollars (SD)	32,174 (7552)	31,965 (7336)	32,056 (7432)	32,063 (7228)
**Neighboorhood environment during adolescence**				
Rural, n (%)	218 (46.7)	202 (49.6)	198 (48.9)	203 (48.6)
Urban, n (%)	249 (53.3)	205 (50.4)	207 (51.1)	215 (51.4)
**Ethnic background**				
Caucasian, n (%)	446 (95.5)	388 (95.3)	388 (95.8)	403 (96.4)
Other, n (%)	21 (4.5)	19 (4.7)	17 (4.2)	15 (3.6)
**Adolescent physical activity profile**				
Nonparticipants, n (%)	34 (7.3)	28 (6.9)	30 (7.4)	30 (7.2)
Dropouts, n (%)	143 (30.6)	122 (30.0)	121 (29.9)	127 (30.4)
Active in unorganized PA, n (%)	82 (17.6)	70 (17.2)	68 (16.8)	74 (17.7)
Active in organized PA, n (%)	139 (29.8)	126 (31.0)	123 (30.4)	127 (30.4)
Active in a variety of PA, n (%)	69 (14.8)	61 (15.0)	63 (15.6)	60 (14.4)
**Physical activity in emerging adulthood**				
Reporting no physical activity, n (%)	45 (9.6)	57 (14.0)	86 (21.2)	94 (22.5)
Mean physical activity (SD) among participants reporting some physical activity (MET-min/week)	2873 (2923)	2715 (2998)	3332 (3462)	3428 (3258)
Mean physical activity (SD) among all participants (MET-min/week)	2597 (2905)	2335 (3359)	2618 (3359)	2659 (3206)

**Fig. 1:**
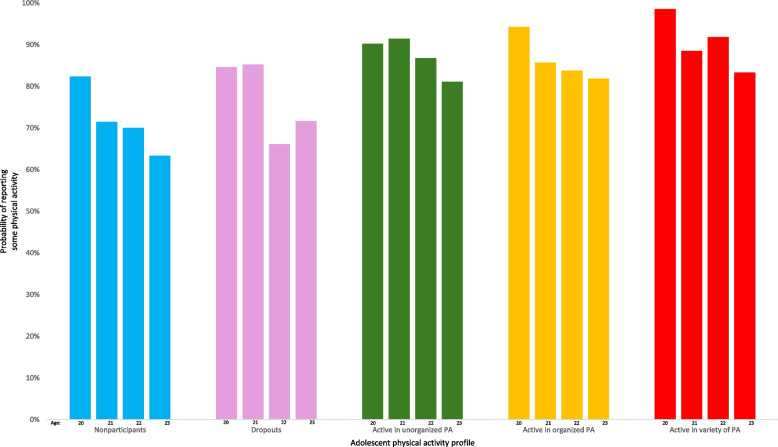
Probability of reporting some physical activity at age 20–23 by adolescent physical activity profile

When modeling the effect of adolescent physical activity profiles on early adults’ physical activity levels, we opted for a correlated model since the covariance was significant (*p* = 0.0004), and statistical indicators (AIC and −2 Log Likelihood) were smaller than for an uncorrelated model. Moreover, the likelihood ratio test was significant and favored the fitted model based on a chi-square test with one degree of freedom (*p* < 0.0001). Whereas participants across all adolescent physical activity profiles demonstrated a general drop in the probability that they report some physical activity during emerging adulthood (Fig. 1), our model highlights that participants who were in one of the three physically active profiles during adolescence had over two times greater odds of reporting some physical activity during emerging adulthood, when compared to those who were not active during adolescence (Table 2). These estimates correspond to medium to large effect sizes, as their associated Cohen’s d coefficients were 0.48 (active in organized physical activity), 0.56 (active in unorganized physical activity), and 0.72 (highly active in a variety of physical activity). When comparing participants who had a dropout physical activity profile during adolescence to those who were nonparticipants, we noted no statistically significant difference in odds of reporting some physical activity during emerging adulthood.

**Table 2:** Adolescent profiles, odds of adult physical activity, and differences in adult physical activity level^1^

	**Occurrence model** (Reporting some vs no MVPA)		**Intensity model** ^2^ (Relative differences in level of MVPA)	
	Logistic regression		Linear regression	
	OR	95% CI	exp (ß)	95% CI
**Adolescent physical activity profile**				
Nonparticipants	Reference		Reference	
Dropouts	1.27	0.52–1.94	1.20	−0.19 – 2.58
Active in unorganized PA	2.75	2.11–3.75	1.74	0.33–3.15
Active in organized PA	2.40	1.67–3.15	1.44	0.05–2.82
Active in a variety of PA	3.66	3.30–5.05	2.15	0.73–3.56
**Variance Components**	Variance	(SE)	Variance	(SE)
Variance(u1)	1	0.29		
Variance(u2)			0.41	0.05
Covariance(u1,u2)			0.32	0.09
^1^Each part of the model includes adjustments for time (data collection cycle), gender, ethnic background, rurality, and income during adolescence ^2^The intensity model includes only emerging adults who reported some physical activity participation *MVPA *Moderate to vigorous physical activity, *OR *Odds ratio, *CI *Confidence interval, *PA *Physical activity, *SE *Standard error				

**Fig. 2:**
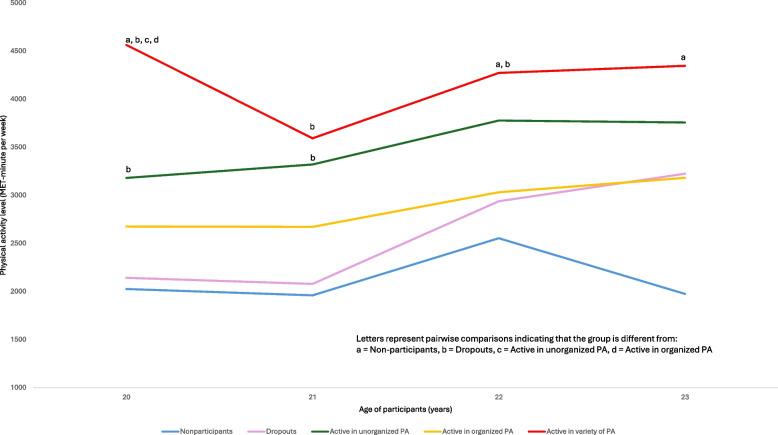
Average physical activity level of active adults according to their adolescent physical activity profiles

Because the outcome (MVPA) in the linear part of the model was log transformed, we exponentiated the regression coefficients to ease interpretations. These values can therefore be interpreted as differences in the ratio of the means of the original outcome (i.e., untransformed MVPA). Specifically, the model suggests that, on average throughout emerging adulthood, and relative to those who were nonparticipants during adolescence, emerging adults who were active in unorganized physical activity during adolescence presented 74% higher levels of MVPA, those who were active in organized physical activity during adolescence were 44% more active, and those who were active in a variety of physical activity during adolescence were 115% more active in emerging adulthood (Table 2). The Cohen’s f^2^ of 0.18 for this model suggests a medium effect of adolescent physical activity profiles for predicting physical activity level in emerging adulthood. Moreover, to complement these main results, pairwise comparisons of participants at specific survey cycles allowed to observe that participants who were highly active in a variety of activities during adolescence reported higher physical activity levels than participants in all other adolescence physical activity profiles at age 20 (Fig. 2). Participants with this profile also reported more physical activity than the dropout profile at age 21 and 22, and more physical activity than the nonparticipants profile at age 22 and 23. When they were age 20 and 21, participants who had been characterized as active in unorganized physical activity during adolescence presented higher physical activity levels than those in the dropout profile. Except for the group of participants characterized as active in unorganized physical activity during adolescence, the average physical activity level of adults in other groups was the lowest at age 21, which coincides with the survey cycle that occurred during the most stringent period of the Covid-19 pandemic related containment restrictions for region [32].

## Discussion

The current analysis highlights that the types of physical activities which characterized the physical activity behaviors of adolescents are important predictors of physical activity in emerging adulthood. More specifically, our results align with conceptual models [33, 34] suggesting that taking part in a greater variety of physical activity during adolescence is associated with being among the most active in emerging adulthood. This analysis extends previous work indicating that variety in physical activity is a positive predictor of future physical activity levels [35, 36] by allowing to understand the contributions of different contexts of physical activity. Specifically, our results show that participation in any type of physical activity during adolescence is associated with a greater likelihood of being active in emerging adulthood, but also that participation in unorganized physical activities during adolescence might provide longer-lasting benefits than involvement in organized physical activities. These associations may nevertheless vary as emerging adults get older since some of our secondary analyses provided suggestions that between group differences in adult physical activity level change from year to year. Further, our results also suggest that even if they have experienced participation in some physical activity during adolescence, emerging adults whose adolescent physical activity profile was characterized by a drop in physical activity report equally low levels of physical activity as those who were consistently not physically active during adolescence.

Crude analyses in this study allowed to identify that, within our sample, there was an age-related increase in the proportion of emerging adults reporting no physical activity. In contrast, we also observed that among those who reported some physical activity, the average level of physical activity they reported increased over time. It is possible that these two results are interdependent whereby it is mostly individuals with the lowest levels of physical activity in the first years of emerging adulthood who reported no physical activity in the later year. This would have the effect of displaying a higher average level of physical activity among those who continue to report some physical activity.

Several conceptual models describing how common pathways of physical activity and sport participation evolve over the life course propose that engaging in a variety of different physical activities during childhood and adolescence puts individuals on a path for being physically active later in life [14]. Consistent with this and a recent study documenting that participating in a greater variety of physical activity is a predictor of higher physical activity levels in the future [37], the current analysis further demonstrates that the characteristics of physical activity participation during adolescence, including a consideration for the contexts in which physical activity is practiced, play a deterministic role in identifying the extent to which emerging adults are physically active. Many mechanisms may be at play to explain this association. First, it is possible that engaging in a wider variety of physical activity during the developmental years of childhood and adolescence results in greater odds that participation in at least one activity be maintained over time. This would be supported by recent evidence demonstrating that people are more likely to take up a specific activity in the future if they have been exposed to this activity earlier in their life [6]. Second, it is also possible that taking part in a greater variety of physical activity types contributes to improving one’s mastery of fundamental movement skills. Explicitly, it was previously demonstrated that physical activity variety is associated with greater improvement in fundamental movement skills [38] and that better fundamental movement skills relate to higher physical activity levels up to six years later [39, 40]. This is natural as sustaining a physically active lifestyle is dependent on experiencing a sense of competence while practicing the activity [41, 42]. Third, the fact that engagement in unorganized physical activity during adolescence was more strongly associated with higher physical activity levels in emerging adulthood than engagement in organized physical activity may also suggest that taking part in a greater variety of physical activity, particularly activities that individuals need to self-organize, promotes the resilience and organizational skills required for individuals to create their own opportunities to be physically active in the long term. Such skills would be particularly beneficial for the maintenance of physical activity during emerging adulthood given it is a period characterized by a decline in opportunities to participate in readily available organized activities. Individuals who have had a chance to develop a better capacity to adapt and to self-control through their prior experiences with unorganized physical activity may therefore have higher odds of being physically active in the future [43, 44]. Fourth, consistent with the concept of positive habit maintenance [45], an alternative hypothesis is that a physically active lifestyle may simply be easier to sustain when fewer barriers threaten to interrupt the behavior. In this context, individuals used to engage in unorganized physical activity may face fewer barriers since opportunities to participate in this type of activity does not decline as much during emerging adulthood as opportunities for participating in organized activities. In relation to this, it is also noteworthy that for almost all groups, the average level of physical activity of active emerging adults was lowest during the peak of the Covid-19 pandemic-related restriction measures. One exception to this was the group of participants characterized as active in unorganized physical activity during adolescence. It is possible that the drop in physical activity level experienced by the other groups was related to a decline in opportunities to practice organized physical activity during the peak of the Covid-19 pandemic risk management contingency plans. It is possible that participants who had more experience with unorganized physical activity were better prepared to succeed in maintaining an active lifestyle in a context where it was often the only type of physical activity that could be practiced. In this way, the Covid-19 pandemic may have had the advantage to clarify that part of the drop in physical activity that is typically associated with transitioning into adulthood could be attributable to fewer opportunities to practice some of the activities one was used to taking part into during adolescence.

Based on these results, interventions to promote long term participation in physical activity should promote a mix of organized and unorganized physical activity during adolescence. Whereas it is generally expected to consider organized forms of physical activity such as sports, exercise classes, and guided sessions whenever documenting youth physical activity [46, 47], the search for strategies to get youth physically active need to ensure there are opportunities for engaging in unorganized physical activities. More specifically, and aligned with the interpretations above, interventions that could result in the maintenance of a physically active lifestyle may include ones that promote exposure to a large variety physical activities during the development years, that facilitate the development of all fundamental movement skills, that provide opportunities to gain organizational skills, and that help the development of positive habits that can be sustained in the long term. Research on the effectiveness of such strategies should be pursued.

Important strengths of this study include the 14 years of follow up of participants with frequent assessments of physical activity behaviors through periods known to be characterized by marked changes in physical activity participation. Moreover, our characterization of adolescents’ physical activity profiles accounted for multiple characteristics of physical activity participation, including MVPA, number of organized physical activity and of unorganized physical activity. Nevertheless, it is possible that other characteristics would also have been important to capture, including the intensity of activities and the social contexts in which they are practiced. It is also possible that measures included in the analysis were subject to over or under-estimation as they were based on self-report. Also, although we were rigorous in our approach for identifying covariates to adjust for in the analysis, there are factors for which we could not account as we did not have data to represent them. Further, although our sample size was adequate to run the main analyses, it is possible that there was low statistical power for some of the pairwise comparisons included to complement Fig. 2. Finally, despite efforts to recruit a sample of participants that included a mix of urban/rural locations from a variety of socioeconomic statuses, the current results may not be generalizable to other populations.

## Conclusions

In conclusion, the current analysis documents that profiles of physical activity participation during adolescence represent strong predictors of physical activity in emerging adulthood. The promotion of participation in various types of physical activity during adolescence, particularly unorganized physical activity, appears crucial for improving odds that people engage in physical activity once they attain emerging adulthood.

## Supplementary Information

Supplementary Material 1

## Data Availability

Data from the MATCH study are available through a data sharing agreement as detailed at: https://impactslab.com/en/research/.

## Abbreviations

BIC: Bayesian Information Criterion
DAG: Directed acyclic graph
IPAQ-SF: International Physical Activity Questionnaire – Short Form
MATCH: Monitoring Activities of Teenagers to Comprehend their Habits
MVPA: Moderate-to-vigorous physical activity
PA: Physical Activity
MET: Metabolic Equivalent of Task
