# Supplementary figures and images for: Adolescent physical activity profiles as determinants of emerging adults’ physical activity
Source: Int J Behav Nutr Phys Act. 2025 Mar 25;22:35. doi: 10.1186/s12966-025-01732-9 (PMC11934583; doi:10.1186/s12966-025-01732-9)

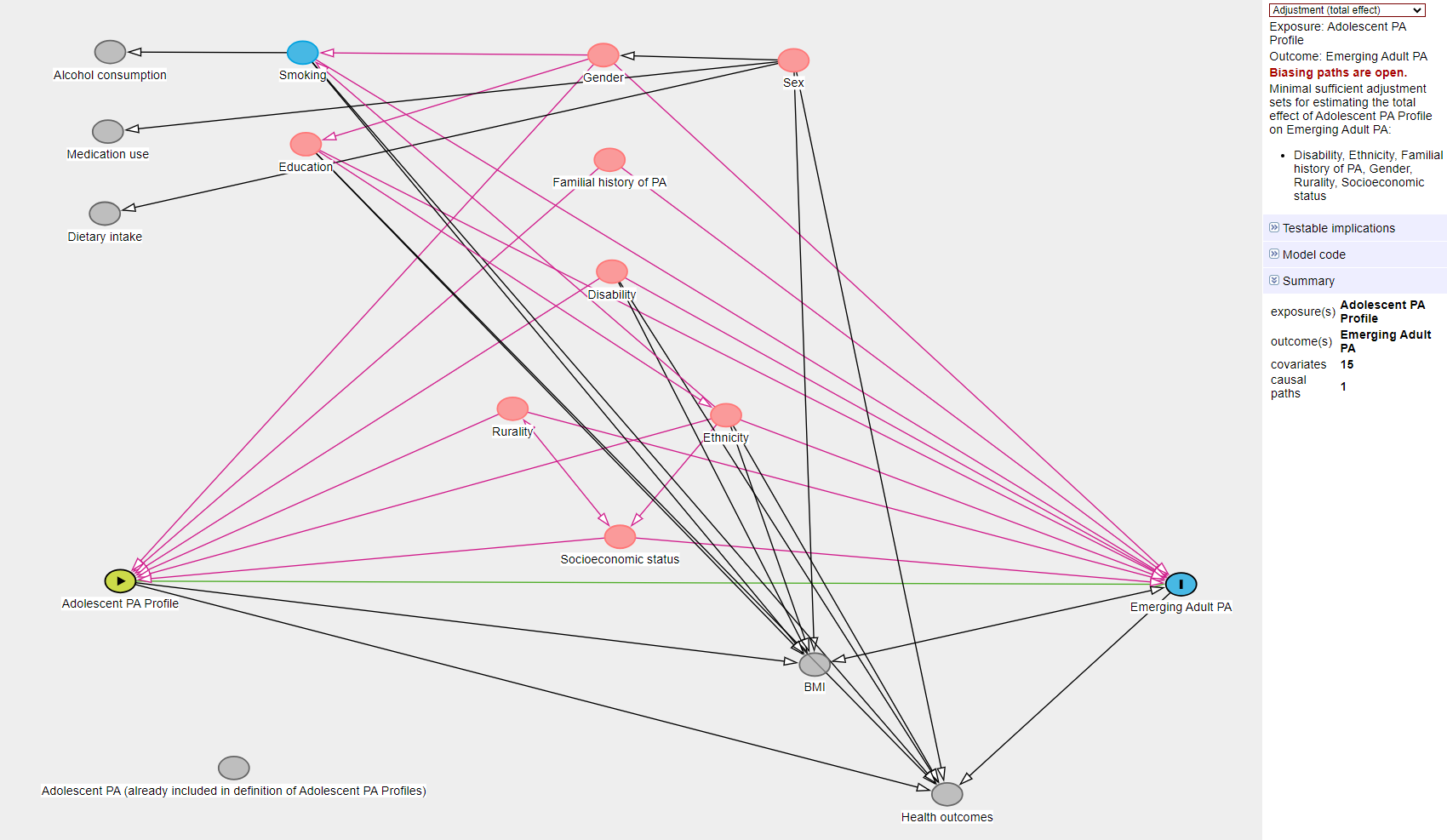

Supplement: Supplementary file 1 — Supplementary Material 1 [file 12966_2025_1732_MOESM1_ESM.png]
